# Baltimore oral epidemiology, disease effects, and HIV evaluation study (BEEHIVE) study protocol: a prospective cohort study

**DOI:** 10.1186/s12903-024-04200-1

**Published:** 2024-04-10

**Authors:** Darien J. Weatherspoon, Gregory D. Kirk, Damani A. Piggott, Vivek Thumbigere-Math, Bruce A. Dye, Mark D. Macek

**Affiliations:** 1 University of Maryland School of Dentistry, 650 West Baltimore Street, Room 2209, Baltimore, MD 21201 USA; 2 Johns Hopkins Medicine, 615 North Wolfe Street, Room E6533, Baltimore, MD 21205 USA; 3 Johns Hopkins Medicine, 600 North Wolfe Street, Baltimore, MD 21287 USA; 4 University of Colorado School of Dental Medicine, Mail Stop F843 13065 East 17th Avenue, Room 104L, Aurora, CO 80045 USA; 5 University of Maryland School of Dentistry, 650 West Baltimore Street, Room 2207, Baltimore, MD 21201 USA

**Keywords:** Human immunodeficiency virus, Oral health, Injection drug use, Non-communicable diseases

## Abstract

**Background:**

As antiretroviral therapy has become widely available and highly effective, HIV has evolved to a manageable, chronic disease. Despite this health advancement, people living with HIV (PLWH) are at an increased risk for age-related non-communicable diseases (NCDs) compared to HIV-uninfected individuals. Similarly, PLWH are at an increased risk for selected oral diseases. PLWH with a history of injecting drugs experience an even greater burden of disease than their counterparts. The overall objective of the Baltimore Oral Epidemiology, Disease Effects, and HIV Evaluation (BEEHIVE) study is to determine the combined effects of HIV infection and NCDs on oral health status. The specific aims of the study are to: (1) determine to what extent HIV status influences access to and utilization of oral health care services; (2) determine to what extent HIV status affects self-reported and clinical oral health status; (3) determine to what extent HIV status influences the progression of periodontitis; and (4) determine to what extent HIV status impacts the periodontitis-associated oral microbiome signature.

**Methods:**

The BEEHIVE study uses a prospective cohort study design to collect data from participants at baseline and at a 24-month follow-up visit. Data are collected through questionnaire assessments, clinical examinations, and evaluation of oral microbiological samples to determine the drivers of oral disease among a high-risk population of PLWH with a history of injection drug use and prevalent comorbid NCDs. The established AIDS Linked to the Intravenous Experience (ALIVE) cohort serves as the source of participants for the BEEHIVE Study.

**Discussion:**

Upon completion of the BEEHIVE study, the knowledge gained will be important in informing future clinical and preventive interventions that can be implemented into medical and dental practice to ultimately help eliminate long-standing oral health inequities that PLWH experience.

## Background

The Baltimore Oral Epidemiology, Disease Effects, and HIV Evaluation Study (BEEHIVE) is a five-year prospective cohort study funded by the National Institutes of Health (NIH), National Institute of Dental and Craniofacial Research (NIDCR), that aims to address current knowledge gaps by advancing scientific evidence on the oral health status among people living with Human Immunodeficiency Virus (HIV) and the bidirectional effects of HIV, oral health, and non-communicable diseases (NCDs). The long-term goal of this study is to advance clinical and public health practice by informing novel approaches for treatment and prevention of oral diseases among people living with HIV (PLWH), and to reduce oral health inequities.

As antiretroviral therapy (ART) has become widely available and a highly effective treatment for HIV, the disease has evolved to a manageable, chronic disease [1, 2]. As a result, improved survival has been observed, with PLWH now having near-normal life expectancy when their disease is appropriately treated and managed [3, 4]. Despite this health advancement, PLWH are at an increased risk for age-related chronic diseases and conditions, including certain cancers, lung diseases, liver diseases, cardiovascular diseases, and cognitive impairment [5, 6]. Disparities in NCDs experienced by PLWH may be attributed, in part, to a higher prevalence of common age-related chronic disease risk factors, including: physical inactivity, smoking, substance use, poor diet and precipitating social determinants, as well as chronic co-infections and adverse ART effects [5]. The direct effects of HIV pathophysiology, including chronic immune dysregulation and systemic inflammation, also may contribute to the increased risk of age-related NCDs in PLWH [7, 8].

In addition to being at greater risk for age-related NCDs, PLWH are at greater risk for selected oral diseases compared to HIV-uninfected individuals as they age [9–11]. PLWH face substantive barriers to accessing oral healthcare due to having fewer financial resources, lacking dental insurance, perceiving stigma, and lacking dental providers with experience and comfort treating PLWH [12]. Additionally, ART use can lead to side effects such as dry mouth, which can in-turn, increase the risk of dental caries, periodontal disease, and oral-associated fungal infections [12]. Given the inextricable connection between oral and systemic health, poorly controlled oral diseases and conditions can contribute to poor NCD-related health outcomes among PLWH [13–15].

An emerging factor contributing to increased risk for oral diseases among PLWH that warrants further research, is the impact of HIV on the oral microbiome and subsequent progression of oral diseases, such as dental caries and periodontitis [16, 17]. The oral microbiome is a complex environment that can play a critical role in promoting either health or disease in the oral cavity [14, 15]. The oral microbiome promotes oral health by maintaining homeostasis, regulating immunity, and preventing colonization of pathogens within the oral cavity [17–19]. However, dysbiosis of the oral microbiome occurs when there are alterations in the diversity, composition, and function of the oral microbiome compartment [20]. In oral diseases such as periodontal disease, oral dysbiosis creates this imbalance, resulting in a predominance of periodontal pathogens, which serves as a precursor to inflammatory processes that cause disease [16, 20–24]. Inflammation is central to overall HIV pathophysiology, and emerging data suggests that HIV promotes microbial dysbiosis, which can result in systemic inflammation that increases the risk for comorbidities experienced by PLWH [25–28]. Therefore, HIV infection could contribute to oral dysbiosis, and this mechanism could help explain the greater risk for oral diseases observed among PLWH [29]. However, more research is needed to understand the specific mechanisms by which HIV infection modulates oral microbiome shifts in pro-inflammatory oral disease, such as periodontal disease.

Importantly, certain segments of the HIV-infected population experience even greater health inequities than other HIV-infected individuals [30–34]. Namely, PLWH who are racial/ethnic minorities, of lower socioeconomic status (SES), and who inject drugs (PWID) all experience a greater burden of disease than their counterparts [30–34]. Therefore, using a multi-level approach to understand the association between multi-level factors and oral health is necessary for informing interventions to eliminate inequities in oral health status among underserved PLWH.

The BEEHIVE study will use questionnaire assessments, clinical examinations, and evaluation of oral microbiological samples to determine the drivers of oral disease among a high-risk population of PLWH with a history of injection drug use and prevalent comorbid NCDs. The long-term goal of this study is to contribute to improving the oral health status and delivery of oral health care to underserved PLWH.

## Methods/design

### Study aims

The overall objective of the BEEHIVE Study is to determine the combined effects of HIV infection and NCDs on oral health status, including their influence on the oral microbiome and subsequent oral disease progression. The specific aims of the study are to: **Aim (1)** Determine to what extent HIV status influences access to and utilization of oral health care services, independent of relevant covariates; and define how the presence and management of comorbid NCDs may act as effect modifiers of the HIV and oral health utilization relationship; **Aim (2)** Determine to what extent HIV status affects self-reported and clinical oral health status; and define how the presence and management of comorbid NCDs may act as effect modifiers of the HIV and oral health status relationship; **Aim (3)** Determine to what extent HIV status influences the progression of periodontitis; and define how the presence and appropriate management of comorbid NCDs may act as effect modifiers of the HIV and periodontitis relationship; and **Aim (4)** Investigate how the periodontal disease-associated oral microbiome signature is influenced by HIV status, controlling for relevant covariates.

### Setting and study design

The established AIDS Linked to the Intravenous Experience (ALIVE) cohort serves as the source of participants for the BEEHIVE Study, with those ALIVE participants consenting to participate representing the BEEHIVE study population. The ALIVE Study, funded through NIH, is one of the largest and longest running prospective cohorts of PLWH and PWID.

The ALIVE study has prospectively followed HIV-infected and HIV-uninfected individuals with a history of injection drug use in a community-recruited cohort in Baltimore since 1988. Importantly, ALIVE participants are not recruited or followed based on their engagement in HIV clinical care or with substance abuse treatment. At semi-annual visits, ALIVE participants complete standardized questionnaires and undergo clinical examination. Detailed information obtained at each follow-up visit includes socioeconomic, behavioral, and clinical parameters for the prior 6-month period. At each visit, HIV-uninfected persons have antibodies to HIV-1 assayed by enzyme-linked immunosorbent assay, with Western blot confirmation. CD4 cell counts are measured on HIV-infected persons at each visit using flow cytometry, and plasma HIV-1 RNA levels determined using reverse-transcriptase PCR methods. Since its establishment, the ALIVE study has characterized the natural history of HIV among PWID, and quantified risk and identified risk factors for comorbid NCDs and mortality among PLWH and PWID [35–42].

ALIVE participants attending their scheduled study visits are recruited to participate in the BEEHIVE study. The BEEHIVE study protocol was reviewed and approved by the Johns Hopkins School of Public Health Institutional Review Board. All BEEHIVE participants provide written informed consent. Participants are eligible for the BEEHIVE study unless: they have fewer than six natural teeth, have notable hearing or vision limitations (participants must be able to hear interview questions, read 16-point text, and see photographs/diagrams of oral conditions), have a history of bacterial endocarditis, or meet the criteria for requiring antibiotic prophylaxis for dental procedures. All data collection and study-related activities take place at the Wood Clinic in east Baltimore, Maryland.

The BEEHIVE Study uses a prospective cohort study design to collect data from participants at baseline and at a 24-month follow-up visit. Study data are collected by licensed dental practitioner members of the research team who have been trained and calibrated on study protocols and data collection procedures. At baseline, participants complete a questionnaire addressing healthcare utilization, self-reported oral health status, dental insurance status, dental beliefs and attitudes, and oral health literacy. Participants also receive an oral screening examination at the baseline visit, assessing number of teeth present, dental caries experience, and periodontal measurements (pocket depth and loss of periodontal attachment). During the follow-up visit, participants will complete a brief questionnaire concerning utilization and oral health status. They will also receive a follow-up screening examination to assess changes in dental caries and periodontal health outcomes.

A sub-set of BEEHIVE Study participants will also provide biospecimens during the follow-up visit. This set of participants will include three groups: (1) PLWH who are well controlled on antiretrovirals, (2) PLWH who are not well controlled, and (3) those who are HIV-negative. These three groups will be cross-linked against their periodontal health status: (1) none/mild/moderate and (2) severe. Therefore, nine groups will provide biospecimens consisting of saliva, buccal epithelial cells, and sub-gingival plaque pooled from the four deepest pockets.

BEEHIVE Study participants are identified by unique study numbers that allow linkage with their ALIVE Study data, thereby allowing for the assessment of important health related NCD covariates. To minimize loss to follow-up, participants are contacted and sent reminders about their 24-month follow-up visit well in advance of their appointment date.

### Characteristics of participants

This report describes the initial 180 participants who were recruited and enrolled into the BEEHIVE study during the first year of data collection (June 2022 to May 2023). Sociodemographic characteristics of the participants are found in Table 1. Approximately, 25% of these participants are HIV-positive. Most BEEHIVE Study participants are male, African American, and of lower SES. Sociodemographic characteristics for HIV-positive and HIV-negative participants in the BEEHIVE Study cohort are similar.

**Table 1:** Sample demographic characteristics of current participants enrolled into the BEEHIVE Study (*N* = 180)*

Characteristic	HIV-Negative 136 (75.6%) n (%)	HIV-Positive 44 (24.4%) n (%)	Total 180 (100%) N (%)
**Age﻿**			
18—64 years	104 (75.9%)	33 (24.1%)	137 (100%)
65 years and older	32 (74.4%)	11 (25.6%)	43 (100%)
**Sex**			
Male	96 (74.4%)	33 (25.6%)	129 (100%)
Female	40 (78.4%)	11 (21.6%)	51 (100%)
**Race/Ethnicity**			
Non-Hispanic Black	109 (73.2%)	40 (26.8%)	149 (100%)
Other	27 (87.1%)	4 (12.9%)	31 (100%)
**Income Last 6 months**			
No income	29 (87.9%)	4 (12.1%)	33 (100%)
Less than $5,000	51 (75.0%)	17 (25.0%)	68 (100%)
$5,000 and greater	56 (70.9%)	23 (29.1%)	79 (100%)
**Marital Status**			
Married or in relationship	27 (87.1%)	4 (12.9%)	31 (100%)
Widowed or divorced	36 (73.5%)	13 (26.5%)	49 (100%)
Never married	73 (73.0%)	27 (27.0%)	100 (100%)
**Receipt of Disability Benefits**			
Yes	**83 (70.3%)**	**35 (29.7%)**	118 (100%)
No	**53 (85.5%)**	**9 (14.5%)**	62 (100%)

* Statistically significant group differences (*p* < 0.05) are listed in **bold** (Chi-Square Test)

### Study procedures

#### Oral health questionnaire assessments

Survey questionnaires are used to obtain data supporting Specific Aims 1–3. The questionnaires were developed from existing and validated surveys [43–45], and include questions about dental visits, insurance, beliefs and attitudes, and oral health literacy. Survey questions are read aloud to participants and responses are recorded on paper forms by trained research staff, with subsequent double-entry into the study database. For some survey items (e.g., those that include multiple-choice options, Likert scales, or pictures), participants are given a flip-chart folder with the questions and response options listed. The surveys take about 12 min to complete. Table 2 summarizes the variables and measures for Specific Aims 1–3 that are obtained using the survey questionnaires.

**Table 2:** Oral health questionnaire-based assessments

Variable/ Type/ Aim(s)	Measures	Variable Format	
		Initial	Final
Access and Utilization of oral healthcare services • Outcome • Aim 1	1. Dental visit (last year) 2. Dental cleaning (last year) 3. Dental insurance (current) 4. Emergency Room visit for dental problem (last 2 years)	1. Multiple Choice 2. Multiple Choice 3. Dichotomous 4. Dichotomous	1. Dichotomous 2. Dichotomous 3. Dichotomous 4. Dichotomous
Oral health status • Outcome • Aim 2	Self-report	Likert Scale (1–5)	Dichotomous
Oral health knowledge, attitudes, and beliefs • Covariates • Aims 1–3	1. Dental beliefs and attitudes 2. Oral health literacy (knowledge)	1. Composite Likert (11–44) 2. Number Correct (0–23)	1. Dichotomous 2. 3 Categories

#### Covariate assessments

Sociodemographic covariates come from the ALIVE Study dataset, and include age, sex, race/ethnicity, household income, presence of medical/dental insurance, marital status, and disability status. NCDs are reflected through various measures of cardiovascular, liver, and kidney health. Data on laboratory and clinical assessments are also available in the analytical data file.

#### Oral health examinations and calibration

The dental practitioner examiners conduct clinical assessments of tooth counts, dental caries experience, and periodontitis, consistent with oral examination procedures used in the National Health and Nutrition Examination Survey (NHANES) [46]. Dental caries assessments are conducted at the tooth- and tooth surface-levels. Periodontal assessments are conducted at four sites per tooth, at each line angle, buccal, and lingual. Table 3 summarizes the clinical outcome variables that are obtained through the oral examination.

**Table 3:** Oral health clinical outcome variables*

Outcome	Assessment	Range of Values	Data Format
Tooth count	Dental mirror	0–28	Count
Overall caries experience teeth (DMFT)/ tooth surface (DMFS)	Dental mirror	0–28/0-128	Counts
Number of actively decayed teeth (DT)/ tooth surfaces (DS)	Dental mirror	0–28/0-128	Counts
Number of missing (due to caries) teeth (MT)/ tooth surfaces (MS)	Dental mirror	0–28/0-128	Counts
Number of filled teeth (FT)/ tooth surfaces (FS)	Dental mirror	0–28/0-128	Counts
% of caries experience due to active decay (teeth/tooth surfaces)	Dental mirror	0-100	Continuous
Dental caries status	Dental mirror	1. Caries free 2. Caries on ≥ 1 tooth	Dichotomous
% of periodontal sites with pockets > 5 mm	Dental mirror/ probe	0-100	Continuous
% of periodontal sites with attachment loss > 4 mm	Dental mirror/ probe	0-100	Continuous
Moderate or severe periodontitis	Dental mirror/ probe	1. Yes 2. No	Dichotomous

*Third molar teeth are not assessed

Prior to study commencement, the dental practitioner examiners were trained and calibrated by an NHANES “gold standard” examiner for tooth count and dental caries experience assessments. A series of calibration training sessions were conducted with a sample of adult participants who were recruited from the University of Maryland School of Dentistry and unaffiliated with the BEEHIVE Study population. Inter-rater reliability (IRR) values were estimated across four examiners using the intraclass correlation coefficient [47, 48]. For the tooth count assessments, the IRR was 100% (Finn coefficient of reliability = 1.0), and the correlation coefficient was statistically significant (*p* < 0.01). The Finn coefficients and associated correlation coefficient p-values for the IRR assessments of dental caries experience are listed in Table 4. Values greater than 0.75 were considered “excellent” [49]. The resulting p-value for the correlation coefficient assessed whether *r* was equal to 0. Values < 0.05 showed that the correlations were statistically significant; that is, examiner scores were highly correlated.

**Table 4:** Inter-rater reliability statistics for assessments of dental caries experience

Assessment	Finn Coefficient	Correlation P-value
Right maxillary DMFT	0.85	< 0.01
Right maxillary DMFS	0.91	< 0.01
Left maxillary DMFT	0.87	< 0.01
Left maxillary DMFS	0.89	< 0.01
Left mandibular DMFT	0.94	< 0.01
Left mandibular DMFS	0.97	< 0.01
Right mandibular DMFT	0.91	< 0.01
Right mandibular DMFS	0.99	< 0.01
Overall DMFT	0.92	< 0.01
Overall DMFS	0.98	< 0.01

DMFT = Mean number of decayed, filled, and missing teeth; DMFS = Mean number of decayed, missing, and filled tooth surfaces

#### Oral microbiome specimen collection

Oral microbiome biospecimens are collected through various methods. Oral buccal mucosal specimen collection is done using a cytobrush (similar to a pap smear brush) rubbed against the inner lining of the cheek to collect cheek mucosal cells and store them for microbial analysis using immunofluorescence. Unstimulated 5mL saliva is collected from participants in falcon tubes, spun down and 1mL supernatant is aliquoted into Eppendorf tubes for (a) microbial analysis and (b) protein analysis. Finally, subgingival dental plaque samples are obtained from participants from their four deepest periodontal pockets using a sterile periodontal curette that is then transferred to sterile saline, and pooled together for microbial analysis. All samples are processed and frozen at -80^o^C until DNA extraction and 16 S rRNA amplicon sequencing is performed.

### Statistical analyses

The extent to which HIV status influences access to and utilization of oral health care services independent of relevant covariates (**Aim 1**), will be assessed through bivariate and multivariable logistic regression analyses. The alpha value for determining statistical significance will be set at 0.01 to account for multiple comparisons. Additionally, effect modification by NCDs will be assessed by including interaction terms in the multivariable model, where applicable.

The extent to which HIV status influences self-reported and clinical oral health status independent of relevant covariates (**Aim 2**), will be assessed using multiple statistical tests. The primary outcome variables will consist of clinical oral health outcome measures that are either dichotomous, count, or continuous variables. Measures of *self-reported oral health status* (dichotomized as “excellent/very good/good” and “fair/poor”) will come from the oral health questionnaire. Measures of *dental caries experience* (dichotomized as “caries-free” vs. “caries-present”) will be obtained from the oral examination. *Periodontal disease status* (dichotomized as “none/mild” and “moderate/severe”) will also be obtained from the oral examination using criteria established by the Centers for Disease Control and Prevention and the American Academy of Periodontology [50]. A variety of analyses, including t-tests, analysis of variance (ANOVA), nonparametric tests (e.g., Mann-Whitney U), zero-inflated negative binomial regression, multiple logistic regression, multiple linear regression, and generalized linear modeling (GLM) will be used to assess the associations between HIV status and the clinical oral health outcome measures depending on the form of the outcome (dichotomous, count, continuous).

The extent to which HIV status influences periodontitis progression independent of relevant covariates will be assessed using various statistical tests (**Aim 3**). The primary exposure variable will be HIV status. The primary outcome variables will be periodontal data measures collected during the follow-up data collection phase. These periodontal outcome variables will have continuous distributions and include *change in % of sites with periodontal pocket depth > 5 mm* and *change in % of sites with periodontal attachment loss > 4 mm*. Paired-sample t-test, repeated-measures ANOVA, and GLM multivariable models will be used to test the relationships between HIV status and these continuous outcomes.

The extent to which periodontal disease-associated oral microbiome signature is influenced by HIV status independent of relevant covariates will be assessed through oral microbiome analyses and subsequent statistical tests (**Aim 4**). Participants will be enrolled sequentially into six groups based on their HIV status (HIV-negative, HIV-positive with suppression, and HIV-positive without suppression) and periodontal disease status (none/mild/moderate, severe) until accrual of 50 participants, per group, is achieved (Table 5). DNA extraction with 16 S rRNA sequencing and whole genome shotgun metagenomics will be performed on obtained oral microbiome samples to assess oral dysbiosis. Principal Components and Bayesian network analyses will be used to assess the association between the oral microbiome and periodontitis status by HIV category. Propensity score matching will be used in longitudinal analyses to assess changes in oral microbiome signatures for participants who transition from periodontally healthy to moderate/severe periodontitis over the two-year follow-up period, compared to those participants who maintain periodontal health.

**Table 5:** Six groups based on Periodontal Disease and HIV status for Aim 4 analyses

Periodontal Disease Status	HIV Status		
	HIV-	HIV + Suppressed	HIV + Without Suppression
None/Mild/Moderate	A1	A2	A3
Severe	B1	B2	B3

A1, A2, A3, B1, B2, B3 = Groups

50 participants per group, *n* = 300

### Sample size and Power

A sample size calculation was performed for Aims 1 and 2. Assuming 80% power and a conservative 0.01 alpha value, 1,200 participants would allow detection of a 10-percentage-point difference in groups (HIV-negative, HIV-positive with viral suppression, and HIV-positive without viral suppression) for dichotomous outcomes (chi-square), and a small effect size difference for mean (t-test, ANOVA) and rank-order (Mann-Whitney U) outcomes. The ALIVE Study has historically had an annual retention rate of nearly 90% [51], therefore, it is expected that 970 individuals will be available for the follow-up phase (analyses conducted in Aims 3 and 4).

A sample size calculation was also performed using less conservative criteria. Assuming 80% power and a 0.05 alpha value, only 340 participants would need to detect a 15-percentage-point difference in groups for dichotomous outcomes, and to detect a medium effect size for mean and rank-order outcomes. Using these less conservative criteria, the ALIVE Study’s 90% retention rate would then yield 306 individuals for Aims 3 and 4.

To calculate the power of detecting an association between overall microbiome profiles across the different HIV and periodontal groups (Aims 3 and 4), R package “micropower” was used [52]. The adjusted coefficient of determination (ω2) was used as a measurement of effect size. A mean and standard deviation of the within-group distance of 0.20 and 0.05 were specified, respectively, and it was determined that 50 participants per group would provide 99.1% power for an ω2 of 0.05.

## Discussion

The major strength of the BEEHIVE Study is its multidisciplinary team consisting of dentists, epidemiologists, physicians, public health practitioners, statisticians, and microbiologists. Equally important to ensuring successful study conduct, is the long-standing relationship with the Baltimore community established through the parent ALIVE Study. Case in point, the ALIVE Study has established trust with the local community, which has resulted in a cohort that has been successfully followed for nearly 30 years, with high annual retention rates. This established trust has helped to establish a community-engaged research partnership between the BEEHIVE Study and its study participants.

Working with a medically vulnerable population in the era of coronavirus disease (COVID-19) requires all necessary safety measures are in place to keep both participants and staff safe. Prior to the BEEHIVE Study launch, time was dedicated to working with study staff to ensure necessary safety and operational protocols were implemented into all study procedures Implementing the BEEHIVE Study within the infrastructure of the long-standing, parent ALIVE Study has helped to minimize potential operational challenges.

In summary, the BEEHIVE Study aims to advance knowledge of the combined effects that HIV status and concomitant NCDs have on oral health status, the oral microbiome, and oral disease progression, among a vulnerable and underserved population, such as PWID. Upon completion of the BEEHIVE Study, the knowledge gained will be important in informing future clinical and preventive interventions that can be implemented into medical and dental practice to ultimately help eliminate long-standing oral health inequities that underserved PLWH experience.

## Data Availability

No datasets were generated or analysed during the current study.

## Abbreviations

AIDS: Acquired Immunodeficiency Syndrome
ALIVE: AIDS Linked to the Intravenous Experience Study
ANOVA: Analysis of Variance
ART: Antiretroviral Therapy
BEEHIVE: Baltimore Oral Epidemiology, Disease Effects, and HIV Evaluation Study
COVID-19: Coronavirus disease
DNA: Deoxyribonucleic acid
GLM: Generalized Linear Modeling
HIV: Human Immunodeficiency Virus
IRR: Inter-rater Reliability
NCD: Noncommunicable Diseases
NIDCR: National Institute of Dental and Craniofacial Research
NIH: National Institutes of Health
PC: Principal Components
PLWH: People Living with Human Immunodeficiency Virus
PWID: People Who Inject Drugs
RNA: Ribonucleic Acid
rRNA: Ribosomal Ribonucleic Acid
